# Effect of Unilateral Acoustic Trauma on Neuronal Firing Activity in the Inferior Colliculus of Mice

**DOI:** 10.3389/fnsyn.2021.684141

**Published:** 2021-06-22

**Authors:** Chun-Jen Hsiao, Alexander V. Galazyuk

**Affiliations:** Department of Anatomy and Neurobiology, Northeast Ohio Medical University, Rootstown, OH, United States

**Keywords:** hyperactivity, bursting, sound exposure, binaural effect, unanesthetized mice

## Abstract

Neural hyperactivity induced by sound exposure often correlates with the development of hyperacusis and/or tinnitus. In laboratory animals, hyperactivity is typically induced by unilateral sound exposure to preserve one ear for further testing of hearing performance. Most ascending fibers in the auditory system cross into the superior olivary complex and then ascend contralaterally. Therefore, unilateral exposure should be expected to mostly affect the contralateral side above the auditory brain stem. On the other hand, it is well known that a significant number of neurons have crossing fibers at every level of the auditory pathway, which may spread the effect of unilateral exposure onto the ipsilateral side. Here we demonstrate that unilateral sound exposure causes development of hyperactivity in both the contra and ipsilateral inferior colliculus in mice. We found that both the spontaneous firing rate and bursting activity were increased significantly compared to unexposed mice. The neurons with characteristic frequencies at or above the center frequency of exposure showed the greatest increase. Surprisingly, this increase was more pronounced in the ipsilateral inferior colliculus. *This study* highlights the importance of considering both ipsi- and contralateral effects in future studies utilizing unilateral sound exposure.

## Introduction

Neuronal hyperactivity is present in many brain diseases. In the auditory system it is believed that hyperactivity may underlie both hyperacusis (exaggerated sensitivity to sound) and tinnitus (a phantom sound without an external stimulus) (Gerken, 1996; Salvi et al., 2000; Eggermont and Roberts, 2004; Roberts et al., 2010; Galazyuk et al., 2012; Salloum et al., 2016; Shore et al., 2016). One of the most common causes for the development of hyperactivity in the auditory system is acoustic trauma after sound exposure. It has been shown that sound exposure leads to cochlear damage and subsequent threshold shifts (Liberman and Kiang, 1978; Kujawa and Liberman, 2009). In response to this damage, the central auditory system increases its gain to compensate for the reduced sensorineural input from the cochlea (Salvi et al., 2000; Schaette and McAlpine, 2011; Galazyuk et al., 2012; Auerbach et al., 2014). As a result of this change in gain, hyperactivity develops in the auditory system as well as in non-auditory brain structures. Noise-induced hyperactivity has been described for the cochlear nucleus (Kaltenbach and Afman, 2000; Brozoski and Bauer, 2005), inferior colliculus (Ma et al., 2006; Bauer et al., 2008; Mulders and Robertson, 2013; Ropp et al., 2014), medial geniculate nucleus (Kalappa et al., 2014), and auditory cortex (Syka and Rybalko, 2000), but not necessarily in auditory nerve fibers (Eggermont and Roberts, 2004).

Hyperactivity in the auditory system has been defined as elevated spontaneous firing, increased bursting, and synchronous firing of auditory neurons. These maladaptive changes have been observed at most levels of the central auditory system (for review see Shore et al., 2016). In the dorsal cochlear nucleus (DCN), sound exposure leads to elevated spontaneous activity, increased neural synchrony, and bursting in fusiform neurons (Wu et al., 2016). Similarly, in addition to increased spontaneous firing, abnormally high neural synchrony and bursting were also reported for non-lemniscal regions of the inferior colliculus (Bauer et al., 2008; Mulders and Robertson, 2013) and elevated bursting was reported for the auditory thalamus (Kalappa et al., 2014). Increased spontaneous firing and synchrony were also found in the auditory cortex following sound overexposure (Robertson and Irvine, 1989; Noreña and Eggermont, 2003). Since hyperactivity often links to hyperacusis and tinnitus, deep knowledge about its development is vital to uncover brain mechanisms underlying these disorders.

Development of hyperactivity in the auditory system in general and in particular in the IC following sound exposure is a complex, long lasting, and dynamic process (for review see Zhao et al., 2016). Immediately after exposure, spontaneous firing rates are elevated in DCN and VCN, whereas IC activity remains unchanged. Two weeks later, increased IC activity begins to be detected, along with continuous hyperexcitation in the DCN (Gröschel et al., 2014). At this stage, ablation of the DCN results in major reductions of IC hyperactivity (Manzoor et al., 2012). However, approximately 8 weeks after exposure the hyperactivity in the IC becomes more prominent, stable, and does not change much after cochlear ablation (Mulders and Robertson, 2009, 2011; Robertson et al., 2013). Nevertheless, this does not mean that the hyperactivity in the IC is intrinsic and completely independent of ascending inputs. Even after the 2-month period the cochlear nucleus has been found to continue to convey hyperexcitation to the IC (Manzoor et al., 2012, 2013).

The vast majority of animals’ studies utilize unilateral sound exposure to induce tinnitus in order to preserve one ear for further behavioral hearing and/or tinnitus assessments (for review see Galazyuk and Hébert, 2015). In the auditory system, the majority of ascending fibers cross into the superior olivary complex and then ascend via the contralateral side of the brainstem to the auditory cortex. Therefore, unilateral exposure is expected to induce hyperactivity predominantly on the contralateral side of the auditory neuroaxis above the level of the auditory brainstem. On the other hand, it is well known that a significant number of neurons within the auditory system have crossing fibers at every level of the auditory pathway (Schofield and Cant, 1996a,b). Therefore, all levels of the central auditory system receive and process information from both the ipsilateral and contralateral sides. Indeed, it has been clearly demonstrated that after ablation of the unilateral auditory nerve, such changes are evident not only in the ipsilateral cochlear nucleus where the auditory nerve fibers are terminated, but also in the contralateral cochlear nucleus, which receives normal input from the cochlea (Rubio, 2006; Whiting et al., 2009). These findings challenge the expectation that the most profound changes in neuronal activity should occur in the contralateral side after unilateral exposure. Our recent work provided some evidence that auditory neurons in the ipsilateral IC show a dramatic increase in bursting after a unilateral sound exposure (Longenecker and Galazyuk, 2016). Therefore, it is important to determine and compare the changes in neural firing of contra- and ipsilateral auditory pathways after unilateral sound exposure.

In the present study we exposed mice unilaterally and recorded changes in the spontaneous firing rate and bursting in neurons of the inferior colliculus in unanesthetized mice. These changes were assessed and compared in contralateral and ipsilateral ICs. We found that all exposed mice developed hyperactivity in the IC and this hyperactivity was the most pronounced in the IC region linked to the frequency range of the sound exposure. Although the hyperactivity was present at both the contra- and ipsilateral IC, the most robust changes were observed in the ipsilateral IC. Our findings strongly suggest that future studies on hyperactivity should pay close attention to the side of recording.

## Materials and Methods

### Subjects

A total of 12 CBA/CAJ mice were used in this study (5 mice in the control group and 7 mice in the sound exposed (SE) group). All animals were between 6 and 12 months of age. Mice were housed in pairs within a colony room, with a 12 h light-dark cycle, at 25°C. Animal procedures in this study were approved by the Institutional Animal Care and Use Committee at Northeast Ohio Medical University.

### Sound Exposure

Animals were at least 5 months old at the time of sound exposure. The procedure of exposure was performed under general anesthesia with intraperitoneal injection of a ketamine/xylazine mixture (100/10 mg/kg). Additional injections (50% of the initial dose) were given to mice intramuscularly 30 min after the initial injection to maintain an appropriate level of anesthesia. One octave narrowband noise centered at 12.5 kHz (8–17 kHz) was presented to mice unilaterally for 1 h. The noise was generated by a wave form generator (Wavetek model 395), amplified (Sherwood RX-4109) to 116 dB Sound Pressure Level (SPL), and then played through an open field loudspeaker (Fostex FT17H) in a soundproof camber. The open field loudspeaker was calibrated with a 0.25 inch microphone (Brüel and Kjaer,4135). Before exposure, the left external ear canal of exposed mice was blocked with a foam earplug (3M classic earplugs, 3M company) followed by a Kwik-Sil silicone elastomer plug (World Precision Instruments). This manipulation typically reduces sound level by 30–50 dB SPL (Turner et al., 2006; Ropp et al., 2014).

### Auditory Brainstem Responses (ABR)

Mice were anesthetized with ketamine/xylazine (100 and 10 mg/kg, respectively). ABRs were recorded in response to 5 ms tone bursts (0.5 ms rise/fall time) presented at frequencies of 4, 12.5, 20, 30, and 40 kHz with the sound level ranged from 80 to 10 dB SPL in 10 dB steps using an RZ6 multi-I/O processor (Tucker-Davis Technologies). Tone bursts were delivered at the rate of 50/s through a speaker (LCY K-100 Ribbon Tweeter, Madisound), which was placed 10 cm in front of the animal’s head. ABR thresholds were measured before, directly following, and 1 month after sound exposure. Stainless-steel electrodes (disposable subdermal needle electrode, LifeSync Neuro) were placed subdermally at the vertex (active), the ipsilateral and contralateral mastoids (references), and at the base of animal’s tail (ground). The evoked potentials were amplified (RA4PA MEDUSA Preamp, Tucker-Davis Technologies), filtered (100–3,000 Hz bandpass), and averaged across 300 repetitions. Thresholds were determined by visual examination of the averaged ABR waveforms in response to each frequency and sound level combination.

### Surgery

A total of 8 mice were used for extracellular recordings. Each mouse was anesthetized during surgery by using 1.5–2.0% isoflurane. A midline incision of the scalp was made and the tissue overlying the cranium was removed. Then a small metal rod was glued to the cranium using dental cement (C&B Metabond, Japan). Following at least 2 days recovery, each animal was trained to stay in a holding device in a single-walled sound attenuating room. The holding device consisted of a custom-made small plastic tube and a small metal holder. During electrophysiological recordings, animals’ ears were unobstructed for free-field acoustic stimulation.

### Extracellular Recordings

Recordings were made from both the ipsi- and contra-lateral inferior colliculus relative to the side of exposure in awake mice inside a single-walled sound attenuating chamber (Industrial Acoustics Company, Inc.). Throughout the recording session (3–4 h), the animal was offered water periodically and monitored for signs of discomfort. After a recording session, the exposed skull was covered with a Kwik-Sil silicone elastomer plug (World Precision Instruments) and the animal was returned to its holding cage. Experiments were conducted at least 2 months post exposure in the SE group and recordings were performed every other day for up to 2 weeks, after which the animal was sacrificed with an IP injection of Fatal-Plus. No sedative drugs were used during recording sessions. If the animal showed any signs of discomfort, the recording session was terminated, and the mouse was returned to its cage.

Recording electrodes were inserted through a small hole drilled in the skull and dura overlying the IC. Extracellular single-unit recordings were made with quartz glass micropipettes (10–20 MΩ impedance, 2–3 μm tip) filled with 0.5 M sodium chloride. Electrodes were fabricated using a P2000 horizontal micropipette puller (Sutter Instrument). The electrode was positioned into the drilled hole by means of a precision (1 μm) digital micromanipulator MP285 (Sutter Instrument) using a surgical microscope (Leica MZ9.5). The relative position of each electrode was monitored from the readouts of digital micrometers using a common reference point on the brain surface.

Extracellular recordings were limited to the central nucleus of the IC based on the depth of recordings. Vertical advancement of the electrode was made by a precision piezoelectric microdrive (Model 660, KOPF Instr.) from outside the sound-attenuating chamber. Recorded action potentials were amplified (Dagan 2400A preamplifier), monitored audio-visually on a digital oscilloscope (DL3024, YOKOGAWA), digitized and then stored on a computer hard drive using EPC-10 digital interface and PULSE software from HEKA Elektronik at a bandwidth of 100 kHz.

The search stimulus consisted of a frequency modulated 3–60 kHz sweep (150 ms duration, 65 dB SPL) presented once per second. This train was repeated while the recording electrode was advanced in 2–4 μm steps. The characteristic frequency of recorded neurons was assessed manually by presenting tone pips 100 ms in duration using a wide range of sound frequencies (3-53 kHz, 2 kHz step) and sound levels (20, 30, 40, and 55 dB SPL). The spontaneous firing rate (SFR) was assessed during a 30 s recording window in which no stimulus was presented. The stimulus system contained a free-field loudspeaker (LCY-K100 Ribbon Tweeter, Madisound), an amplifier (HCA-750A, PARASOUND) and a Tucker-Davis Technologies system 3 (RX6 multifunction processor, PA5 programmable attenuator, SigGenRP software, Tucker-Davis Technologies).

### Data Analysis

All statistical analyses were accomplished using GraphPad Prism 8 (version 8.4.3., GraphPad). In ABR data, a two-way ANOVA with a Tukey post-test was used to compare thresholds at the three experimental time points. For the extracellular recording data, a Mann-Whitney test was used to compare the Control and Sound Exposure groups. For multiple comparisons, a Kruskal-Wallis test was used with a Dunn’s *post hoc* test. A simple linear regression was utilized to determine the relationship between the characteristic frequency and recording depth. Data are presented as mean with standard deviation (*SD*) or standard error of the mean (SEM) and *p* < 0.05 criteria was used to determine statistical significance.

## Results

### The ABR Thresholds Were Temporary Shift in the Exposed Ear After Unilateral Acoustic Trauma

**FIGURE 1:**
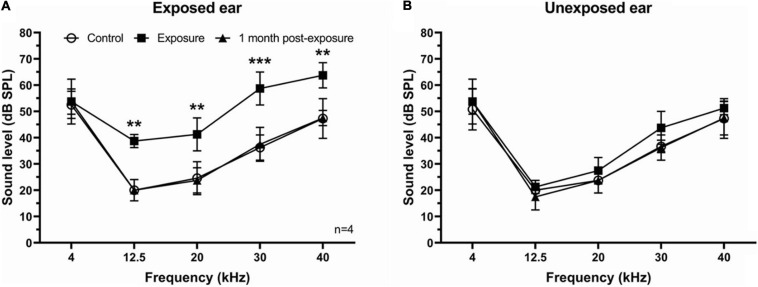
ABR thresholds in SE group. **(A)** ABR thresholds were temporary increased immediately following sound exposure in the exposed ear and recovered back to the control level 1 month post-exposure. **(B)** ABR thresholds showed no difference after sound exposure in the unexposed ear. Mean ± *SD*. Significant changes indicated with (**) at *p* = 0.001 level or (***) at *p* = 0.0001 level.

To assess the effect of unilateral acoustic trauma on hearing, the ABR thresholds were determined in four mice before, directly following, and 1 month post-exposure in both ears. We found a temporary threshold increase at 12.5, 20, 30, and 40 kHz right after sound exposure in the exposed ear which recovered to control levels 1 month later (Figure 1A). In contrast, ABR thresholds in the unexposed (blocked) ear were not affected by sound exposure (Figure 1B).

### Unilateral Acoustic Trauma Increases Spontaneous Firing Rate of IC Neurons

**FIGURE 2:**
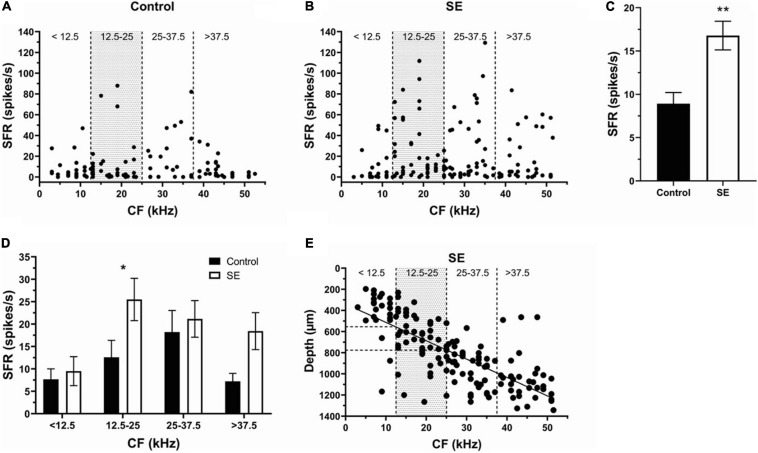
Sound exposure increases SFRs in IC neurons with CFs at or above the center frequency of exposure. **(A,B)** The SFR is plotted against the CF of each neuron in the control (*n* = 107) and SE groups (*n* = 153). **(C)** Mean SFR in the control (8.9 ± 1.3 spikes/s) and SE group (16.78 ± 1.66 spikes/s). **(D)** Mean SFR in the four frequency ranges of control and SE groups. **(E)** The relationship between CF and recording depth of IC neurons in SE group. Mean ± SEM. Significant SFR changes indicated with (*) at *p* = 0.05 level or (**) at *p* = 0.001 level.

To determine the effect of unilateral acoustic trauma on SFR, extracellular single unit responses of 371 neurons were recorded in control (unexposed) and sound exposed mice in contra- and ipsilateral ICs relative to the side of exposure (Figures 2A,B). We found that the mean SFRs of IC neurons in the control group was 8.9 ± 1.3 spikes/s and was no different between right and left ICs (right, 8.9 ± 2.2 spikes/s; left, 8.5 ± 1.5 spikes/s, *p* = 0.61). The average SFR, however, was significantly increased in the SE group compared to control (Figure 2C, control, 8.9 ± 1.3 spikes/s; SE, 16.78 ± 1.66 spikes/s). These changes were not uniformly distributed across neurons with different characteristic frequencies (CFs; compare Figures 2A,B). To determine whether IC neurons within the CF range showed more pronounced increase in SFR, we divided neurons with different CFs into 4 roughly equal frequency ranges (*n* = 48, 79, 73, and 60, respectively). In our study mice were exposed to one octave narrow-band noise (8–17 kHz) with a center frequency of 12.5 kHz. Previous research demonstrated that the neurons most affected by exposure have CFs at or above the center frequency of exposure (Mulders and Robertson, 2009; Longenecker and Galazyuk, 2011; Turner et al., 2012; Coomber et al., 2014; Ropp et al., 2014). Therefore, the frequency range from 0 to 12.5 kHz (all CFs below the center frequency of exposure) defined the size of the frequency step to partition the four ranges (<12.5, 12.5–25, 25–37.5, and >37.5 kHz). In agreement with previous studies, the IC neurons having CFs within the range of sound exposure (12.5–25 kHz) showed the most robust increase in SFR (Figure 2D, Control, 12.59 ± 3.77 spikes/s; SE, 25.49 ± 4.72 spikes/s).

Previous research reveals a tonotopic map in the IC with a spatial gradient of CFs oriented in a dorsolateral (low frequencies) to ventromedial (high frequencies) direction. Consistent with this organization, we found a linear relationship between the CF and recording depth of IC neurons in the SE group (Figure 2E, Y = 17.37^∗^X + 338.7, *R*^2^ = 0.55). These regressions allowed us to identify the depth (550–780 μm) within the IC where the neurons with more pronounced SFR increase were found following a narrow-band (8–17 kHz) noise exposure.

### After Unilateral Sound Exposure Ipsilateral IC Neurons Demonstrate Higher SFR Compared to Contralateral IC

**FIGURE 3:**
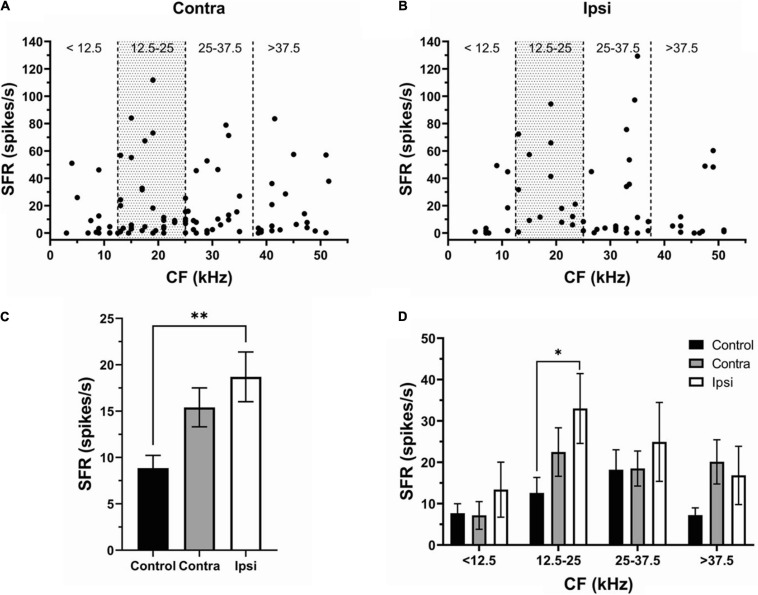
The effect of sound exposure on SFR is more robust in the ipsilateral IC. **(A,B)** The SFR against the CF in neurons of contralateral (*n* = 97) and ipsilateral IC (n = 56). **(C)** Mean SFR in IC neurons of the control (8.9 ± 1.3 spikes/s), contralateral SE (15.41 ± 2.1 spikes/s) and ipsilateral SE (18.7 ± 2.69 spikes/s) groups. **(D)** Mean SFR in control, contra- and ipsilateral SE IC at four frequency ranges (<12.5 kHz; 12.5–25 kHz; 25–37.5 kHz; and > 37.5 kHz). Mean ± SEM. (^∗^) *p* = 0.05 level or (^∗∗^) at *p* = 0.001 level.

To determine whether one side of IC was more affected than the other, we separated and compared neurons recorded in contra- (130 neurons) (Figure 3A) and ipsilateral (93 neurons) (Figure 3B) IC. Although SFR was increased in both contra- and ipsilateral IC neurons, the ipsilateral IC was more affected by exposure (Figure 3C, Control, 8.9 ± 1.3 spikes/s; Contra-, 15.41 ± 2.1 spikes/s; Ipsi-, 18.7 ± 2.69 spikes/s). The SFR increase was most evident in the CF range 12.5–25 kHz of both contra- and ipsilateral IC. This change was statistically significant in ipsilateral (33.01 ± 8.45 spikes/s) but not in contralateral IC (22.48 ± 3.77 spikes/s) compared to controls (Figure 3D).

### Acoustic Trauma Increases Spontaneous Bursting Activity of IC Neurons

**FIGURE 4:**
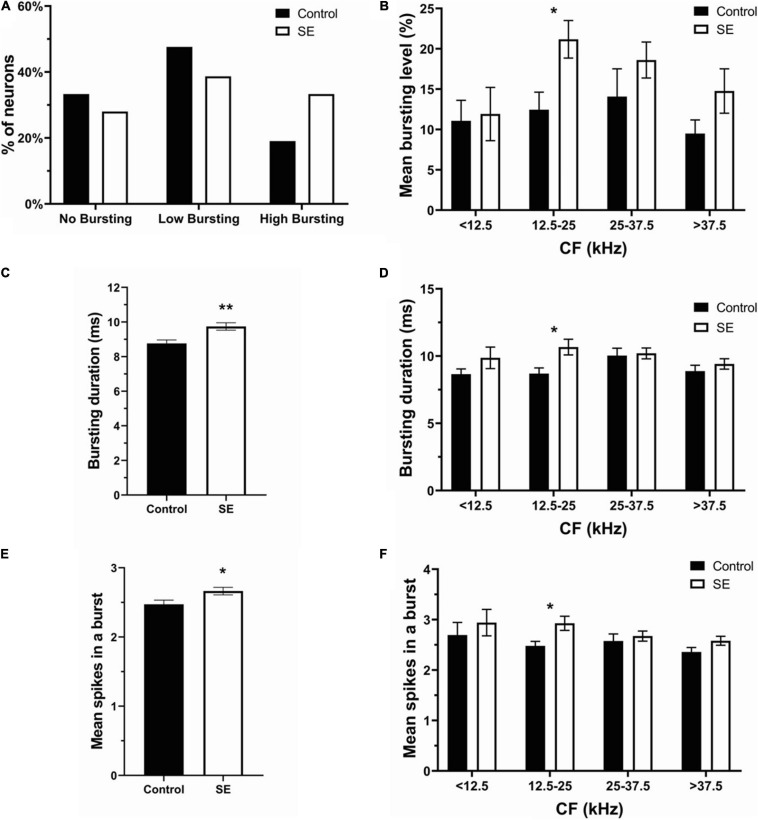
Bursting activity was elevated in the SE group especially within the CF frequency range of 12.5–25 kHz. **(A)** The percentage of no bursting, low bursting and high bursting neurons in control and SE groups. **(B)** Mean bursting level vs. four CF ranges in control and SE groups. **(C)** Bursting duration and **(E)** mean spikes in a burst in control (8.76 ± 0.2 ms; 2.47 ± 0.06 spikes) and SE groups (9.75 ± 0.21 ms; 2.66 ± 0.05 spikes). **(D)** Bursting duration and **(F)** mean spikes in a burst vs. four CF ranges in control and SE groups. Mean ± SEM. (*) *p* = 0.05 level or (**) at *p* = 0.001 level.

To assess the effect of unilateral acoustic trauma on spontaneous bursting activity in IC neurons, we adopted the burst definition from Bauer et al. (2008). To be defined as a bursting event, each burst needed to satisfy the following 6 criteria: (1) maximum allowable burst duration: 310 ms; (2) maximum ISI at burst start: 500 ms; (3) maximum within-burst ISI: 10 ms; (4) minimum interval between bursts: 50 ms; (5) minimum burst duration: 5 ms; (6) minimum number of spikes in a burst: 2. In addition, the present data was arbitrarily divided into three bursting levels: no bursting (NB), low bursting (LB) (< 20%) and high bursting (HB) (≥20%) (Figure 4A). In the control group (147 neurons), 66.67% of neurons showed bursting activity (47.62% LB, 19.05% HB), whereas 33.33% of neurons did not (Figure 4A). In the sound exposed group (224 neurons), 72.0% of IC neurons were classified as bursting while 28.0% of neurons showed no bursting. The bursting neurons (72%) were then divided into low bursting and high bursting groups (38.67 and 33.33%, respectively) (Figure 4A). We found that after sound exposure, the proportion of high bursting neurons was increased while low bursting decreased compared to controls. Further, the mean bursting level was elevated only in the neurons having CFs in the range of 12.5–25 kHz (Figure 4B). Regarding bursting parameters, the SE group showed significant increases in both bursting duration and mean spikes in a burst compared to the control group (Figures 4C,E, control, 8.76 ± 0.2 ms, 2.47 ± 0.06 spikes; SE, 9.75 ± 0.21 ms, 2.66 ± 0.05 spikes). Again, these changes were most evident and significant within the CF range of 12.5–25 kHz (Figures 4D,F).

### Unilateral Acoustic Trauma Differentially Affects Spontaneous Bursting Activity in Contralateral and Ipsilateral IC Neurons

**FIGURE 5:**
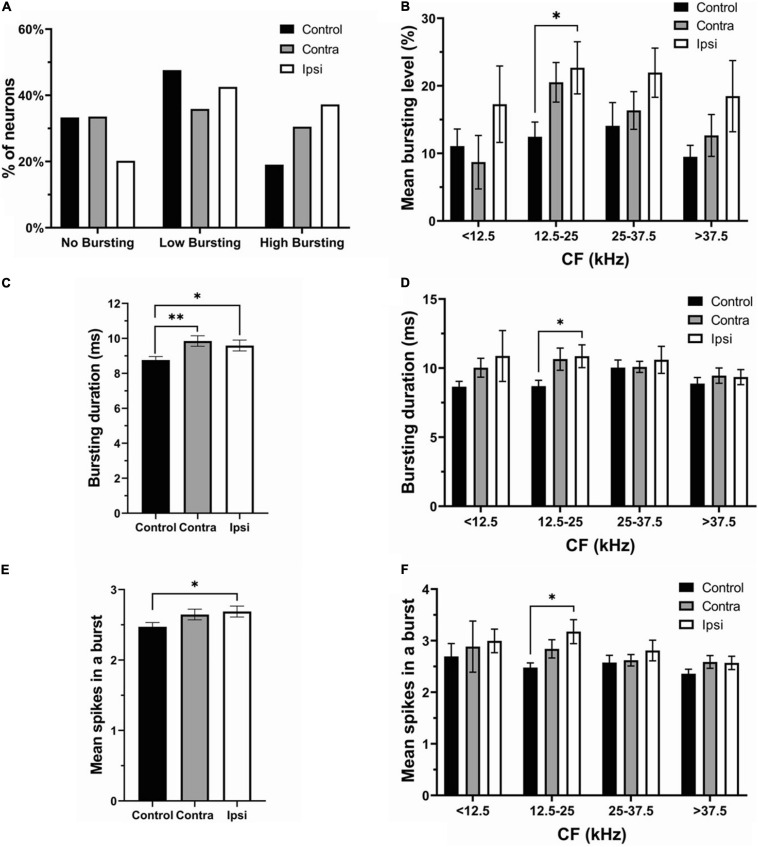
The ipsilateral IC contributed to the changes in bursting activity after unilateral acoustic trauma. **(A)** The percentage of no bursting, low bursting and high bursting neurons in control, contra- and ipsilateral SE ICs. **(B)** Mean bursting level vs. CF range in control, contra- and ipsilateral SE ICs. **(C)** Bursting duration in all recorded neurons in control (both ICs), contra and ipsilateral SE ICs. **(D)** Bursting duration in four different CF frequency ranges. **(E)** Mean spikes in a burst in control (both ICs), contra and ipsilateral SE ICs. **(F)** Mean spikes in a burst in four different CF frequency ranges. Mean ± SEM. (^∗^) *p* = 0.05 level or (^∗∗^) at *p* = 0.001 level.

The proportion of no bursting (control: 49 neurons; contra: 44 neurons; Ipsi: 19 neurons), low bursting (control: 70 neurons; contra: 47 neurons; ipsi: 40 neurons), and high bursting neurons (control:28 neurons; contra: 40 neurons; ipsi: 34 neurons) was differentially altered by unilateral sound exposure (Figure 5A). The percentage of NB neurons in the contralateral IC was similar to controls after exposure, but decreased in the ipsilateral IC (control—33.33%, contralateral—33.59% ipsilateral—20.21%). The proportion of LB neurons was decreased in both ICs in SE mice compared to control mice. However, this decrease was more pronounced in the contralateral IC (control—47.62%; contra–35.88%; ipsi—42.55%). In contrast, the percent of HB neurons increased in both ICs with a larger change in ipsilateral IC (control–19.05%; contra–30.53%; ipsi—37.24%). Although the mean bursting level tended to increase in both contra and ipsilateral ICs in neurons with a wide range of CFs, this increase was most evident in the range of 12.5–25 kHz in the ipsilateral IC (Figure 5B). Similarly, the bursting duration increased in both ICs (Figure 5C), whereas this increase was most noticeable in the range of 12.5–25 kHz in the ipsilateral IC (Figure 5D). The mean number spikes per burst was also slightly elevated in both ICs after sound exposure but was significant in the ipsilateral IC (Figure 5E). In accordance with other parameters of bursting this increase was significant in the ipsilateral IC in the range of 12.5–25 kHz (Figure 5F).

## Discussion

The main goal of this research was to determine changes in firing activity of the auditory midbrain neurons of mice following a unilateral sound overexposure. Changes in spontaneous firing rate and bursting were assessed in contralateral and ipsilateral ICs relative to the side of exposure and then compared. In accordance with previous reports the spontaneous firing rate and bursting were increased in IC neurons by exposure (Ma et al., 2006; Bauer et al., 2008; Mulders and Robertson, 2013; Ropp et al., 2014; Ma et al., 2020). Remarkably, this increase was evident in both the contralateral and ipsilateral ICs, with a more robust effect in the ipsilateral IC.

### ABR Threshold Were Temporary Increase and Recovery in the Exposed Ear

The main goals of ABR testing were to confirm three expected outcomes of sound exposure. First, that our unilateral exposure affected the exposed ear only, with little or no effect on the unexposed ear. Second, that our exposure caused an ABR threshold shift, indicating that the sound exposure was effective in inducing an acoustic trauma. Third, that this threshold shift was temporary, which after several weeks returned to the level before exposure. Therefore, we tested ABR thresholds in the ipsilateral and contralateral ears relative to the side of exposure independently before, immediately after, and 1 month following sound exposure. Our data indicate that all expected outcomes were confirmed.

### Sound Exposure Induces Hyperactivity in the IC

At present, there is an agreement in the field of tinnitus research that hyperactivity in the auditory system is an underlying mechanism of tinnitus. On the other hand, some laboratory animals or human subjects exhibiting hyperactivity do not show tinnitus. Thus, hyperactivity is a necessary, but not a sufficient, condition for a phantom sound percept or tinnitus.

Studies utilizing both tinnitus animal models and human research advocate that hyperactivity in the auditory system often correlates with tinnitus. Although such hyperactivity has been demonstrated for all levels of the central auditory pathway, the most affected specific nuclei differ between studies. Research from multiple labs using different animal models found that hyperactivity in the cochlear nucleus correlates with behavioral evidence of tinnitus (see review Wu et al., 2016). Tinnitus-related hyperactivity in the dorsal cochlear nucleus (DCN) has been associated with reduced inhibition as well as increased excitation from the non-auditory circuitry following cochlear injury (Wang et al., 2009; Middleton et al., 2011; Dehmel et al., 2012; Koehler and Shore, 2013). The presence of hyperactivity in sound exposed animals in the IC is still debated. On one hand, several studies showed that increased SFR occurred in neurons with best frequencies (BFs) overlapping the regions of hearing loss (Ma et al., 2006; Mulders and Robertson, 2009; Longenecker and Galazyuk, 2011; Manzoor et al., 2013) or widespread increases in SFR without frequency specificity (Bauer et al., 2008; Berger et al., 2014; Ropp et al., 2014). On the other hand, one study failed to find a significant change in SFR of IC neurons following a sound exposure in mice (Shaheen and Liberman, 2018).

Our results reveal that hyperactivity is present in the exposed mice. After unilateral exposure, both the SFR and bursting were increased in both the contra and ipsilateral ICs (Figures 2C, 4C,E) and these changes were linked to the frequency range of exposure (Figures 2D, 4D,F). However, the elevation in SFR and bursting was significant only in the ipsilateral IC compared to controls (Figures 3C, 5E). Although not significant, the presence of consistent increases in SFR and bursting in the contralateral IC suggests that these effects would reach statistical significance with a substantial increase in the sample size of recorded neurons. It is possible that the absence of significant changes in hyperactivity in several studies might also be explained by a small sample size and/or the focus on data from the contralateral IC relative to the side of exposure. The fact that we observed this hyperactivity several months after exposure makes us confident that this is a chronic sign of an acoustic trauma.

### The Ipsilateral Dominance in Hyperactivity

The most unexpected finding of this study is that both the contralateral and ipsilateral ICs showed clear signs of hyperactivity, and that the ipsilateral IC was more affected following a unilateral sound exposure. Consistent with these results, an ipsilateral dominance has been reported for elevation of bursting firing in IC neurons after unilateral exposure (Longenecker and Galazyuk, 2016). Despite the consistent trend of ipsilateral dominance in the present study, the difference between ipsilateral and contralateral IC was not statistically significant. These findings are surprising, because unilateral sound exposure is expected to cause damage mostly in the affected ear, as was confirmed by a temporary ABR threshold shift immediately following sound exposure (Figure 1). As a result, the sensorineural hearing loss induced by exposure should lead to a reduction of the sensory input from the cochlea to the central auditory system. To compensate for the loss, the neurons in the cochlear nucleus on the exposed side should increase their activity. This compensatory increase in the central auditory activity in response to the loss of sensory input is referred to as central gain enhancement (see review by Auerbach et al., 2014). Enhanced central gain is hypothesized to give rise to hyperactivity in the central auditory system which is believed to be responsible for development of hyperacusis and/or tinnitus. Hyperactivity has been well characterized for the fusiform neurons of the dorsal cochlear nucleus after sound exposure (Brozoski et al., 2002; Shore et al., 2008; Finlayson and Kaltenbach, 2009; Pilati et al., 2012; Wu et al., 2016). DCN neurons mainly project to the contralateral IC (see review of Cant and Benson, 2003). An ipsilateral projection is described by most authors, although it appears to be small. Therefore, after unilateral sound exposure we should expect hyperactivity mainly to be present in the contralateral IC. In contrast, in the present study we observe hyperactivity in both ICs with ipsilateral dominance. Thus, bilateral projection from affected cochlear nucleus to both ICs after unilateral exposure cannot explain our findings. A possible explanation for this result is that unilateral exposure leads to maladaptive changes in neuronal firing or hyperactivity in both the ipsilateral and contralateral cochlear nuclei, which then project this hyperactivity to both ICs.

### Unilateral Exposure Induces Hyperactivity in Both Cochlear Nuclei and Therefore in Both ICs

In our study there were bilateral changes in the SFR and bursting firing properties of IC neurons. Such changes might be inherited from ascending projections from both ipsilateral and contralateral cochlear nuclei. Unilateral sound exposure could directly alter firing properties of neurons in the ipsilateral cochlear nucleus, one of the main inputs to IC, and also indirectly affect the contralateral cochlear nucleus via crossed connections between the cochlear nuclei (Cant and Benson, 2003). Previous research reveal the likelihood of crossed inhibitory connections between the cochlear nuclei (Cant and Gaston, 1982; Wenthold, 1987; Schofield and Cant, 1996b; Alibardi, 2000). Cant and Gaston (1982) reported connections between the dorsal and ventral cochlear nuclei projecting to the contralateral, anteroventral, and posteroventral cochlear nucleus, as well as to the dorsal cochlear nucleus fusiform cell layer. Schofield and Cant (1996a) described labeled boutons that made contacts in the contralateral fusiform neurons and deep layers of the dorsal cochlear nucleus. Potashner et al. (2000) found that unilateral manipulation of peripheral input altered glycine neurotransmission in both the contralateral and ipsilateral dorsal cochlear nuclei. Therefore, plastic changes in one cochlear nucleus are likely to cause either direct or indirect changes in the contralateral cochlear nucleus. More direct evidence for plastic changes in the contralateral cochlear nucleus to unilateral auditory depravation comes from two studies where changes in glutamatergic synapses were identified in both the affected and unaffected cochlear nuclei (Rubio, 2006; Whiting et al., 2009). In an earlier study the AMPA receptors were found to be redistributed in DCN neurons receiving direct contact from the auditory nerve on the side of the auditory nerve lesion and also in the neurons of the contralateral DCN which receives an intact auditory nerve synaptic input (Rubio, 2006). In a following study, similar bilateral changes were observed in response to a mild (∼20 dB) conductive unilateral hearing loss in rats (Whiting et al., 2009). They detected that auditory nerve synapses on bushy and fusiform neurons of the ventral and dorsal cochlear nucleus, respectively, upregulated the GLU3 AMPA receptor subunit, whereas inhibitory synapses showed decreased expression of the GlyRa1 subunit. These changes, however, were fully reversible once the earplug causing conductive hearing loss was removed. Hence, multiple studies provide evidence for the mechanism by which unilateral acoustic trauma can cause bilateral changes in firing activity of auditory neurons throughout the central auditory system. On the other hand, it is still unclear why in the present study such changes were more evident in the ipsilateral IC. Future research is needed to shed light on this phenomenon.

In summary, the present study has demonstrated that a unilateral acoustic trauma leads to development of hyperactivity in both contralateral and ipsilateral ICs with a greater ipsilateral effect several months after exposure. In both ICs, an increase in SFR and bursting is linked to the frequency range of exposure. These results confirm that an acoustic trauma reliably induces chronic hyperactivity in the auditory midbrain. They also highlight the importance for research on hyperactivity to evaluate the side of recording within the auditory pathway.

## Data Availability Statement

The raw data supporting the conclusions of this article will be made available by the authors, without undue reservation.

## Ethics Statement

The animal study was reviewed and approved by the Institutional Animal Care and Use Committee at the Northeast Ohio Medical University.

## Author Contributions

AG contributed to conception and design of the study and wrote sections of the manuscript. C-JH organized the database, performed the statistical analysis, and wrote the first draft of the manuscript. Both authors contributed to manuscript revision, read, and approved the submitted version.

## Conflict of Interest

The authors declare that the research was conducted in the absence of any commercial or financial relationships that could be construed as a potential conflict of interest.
